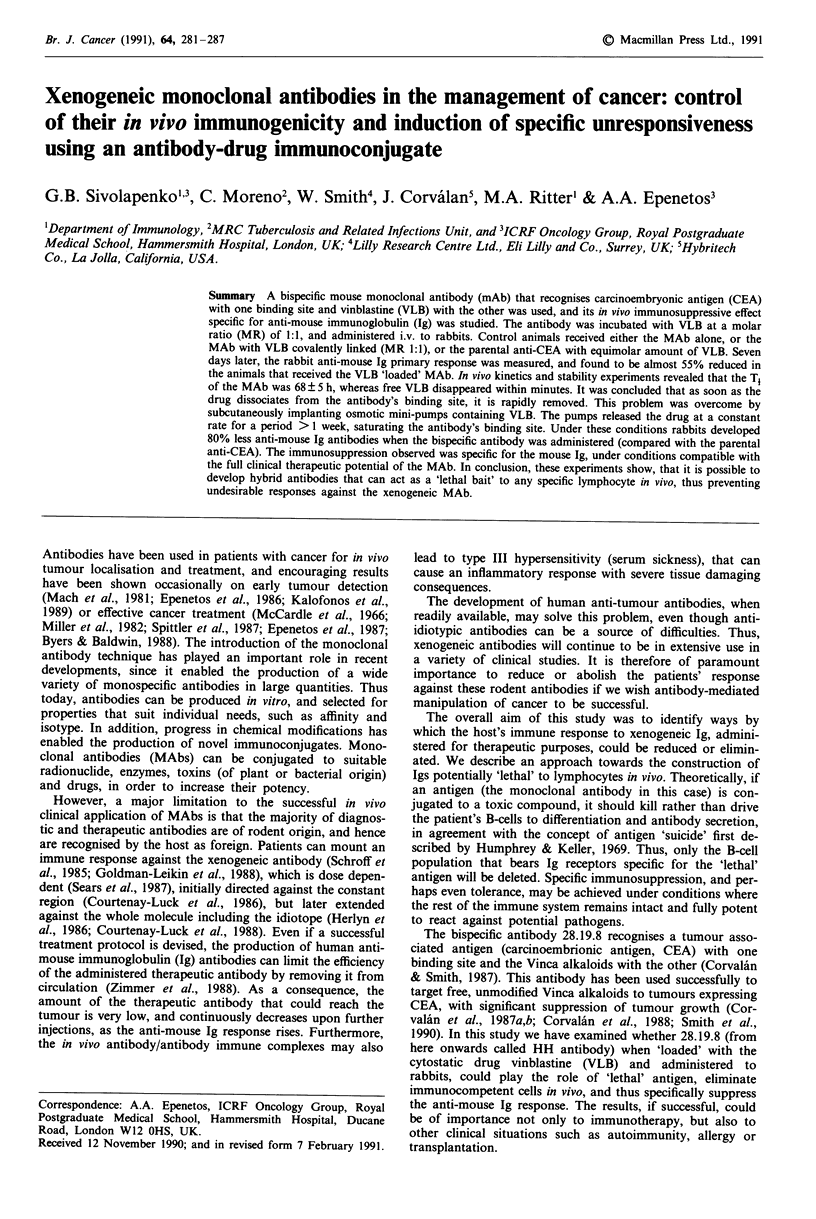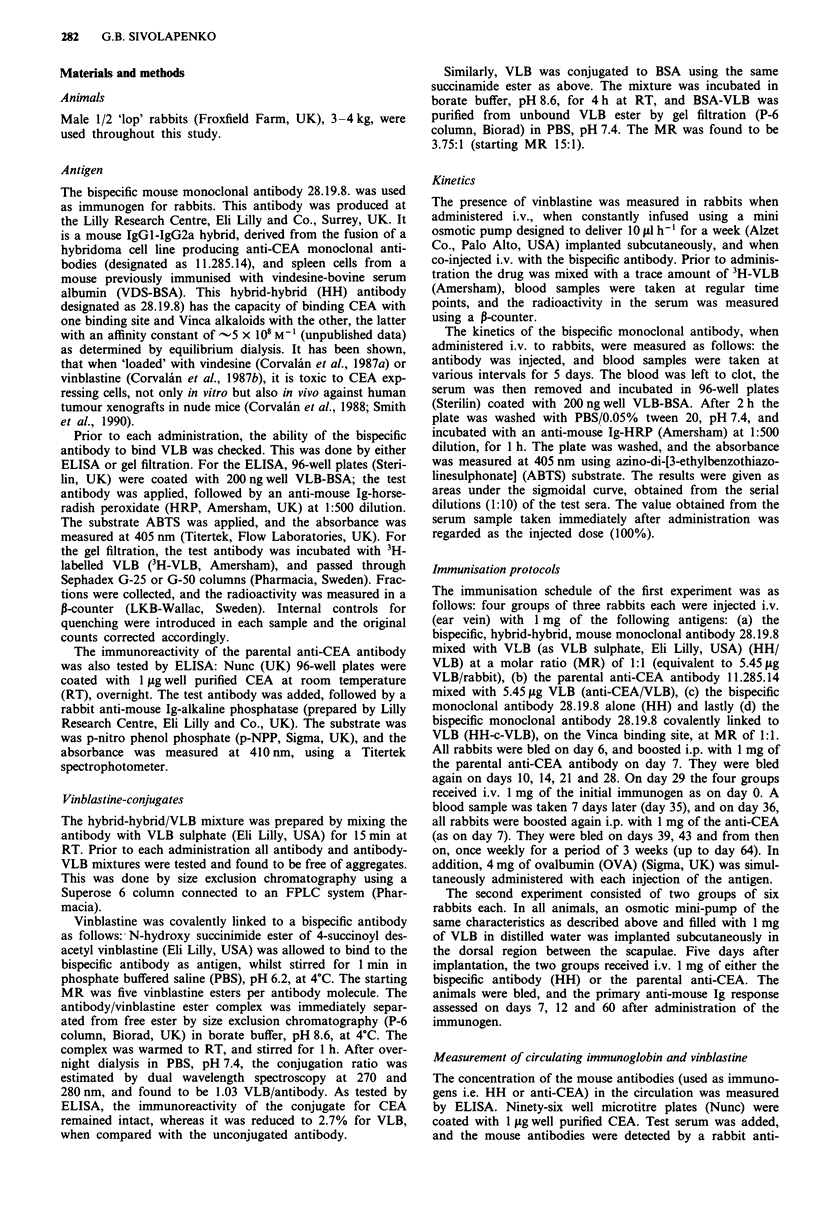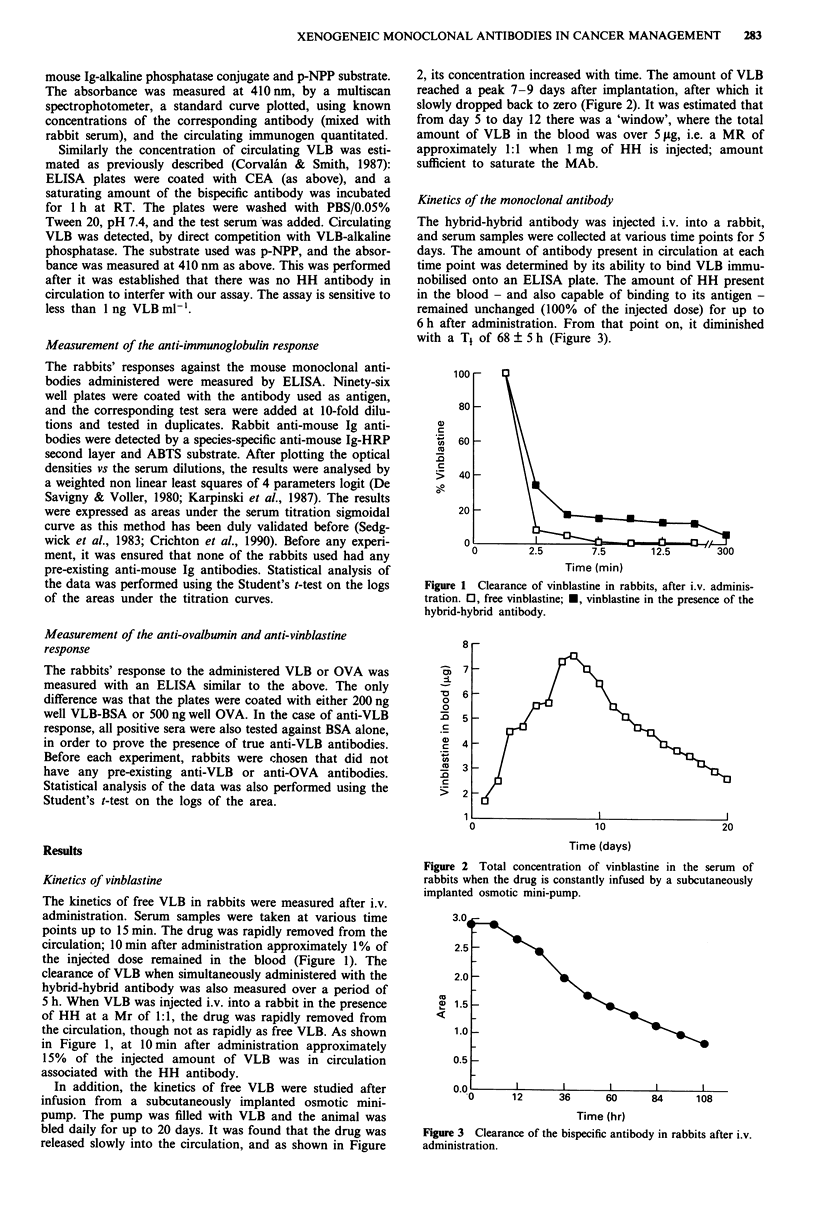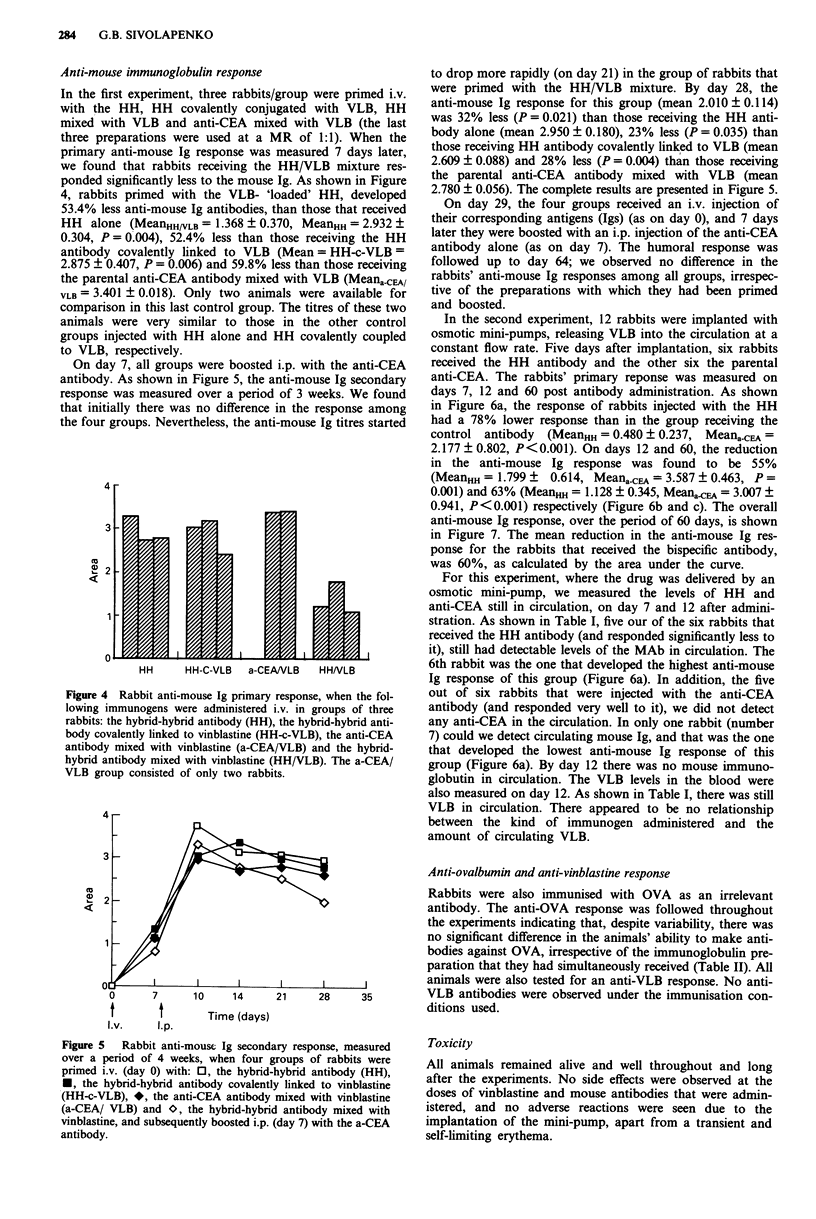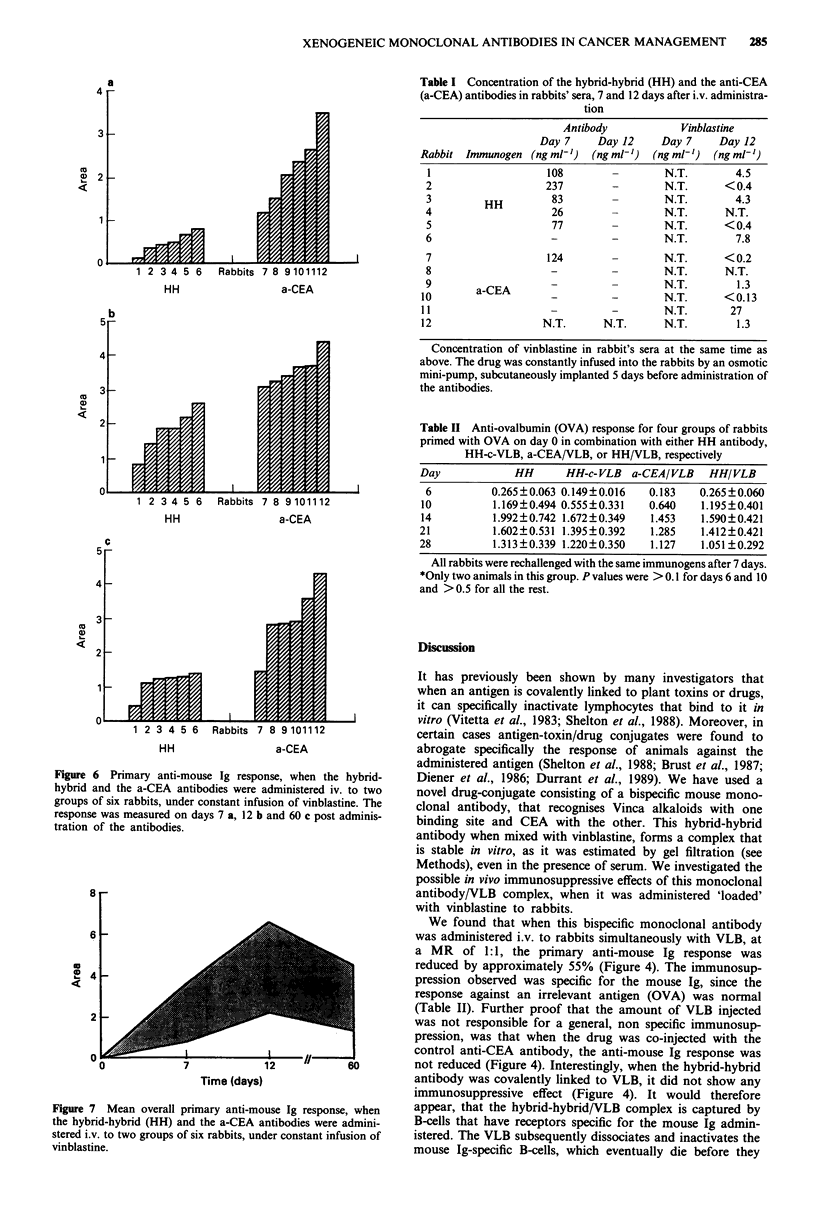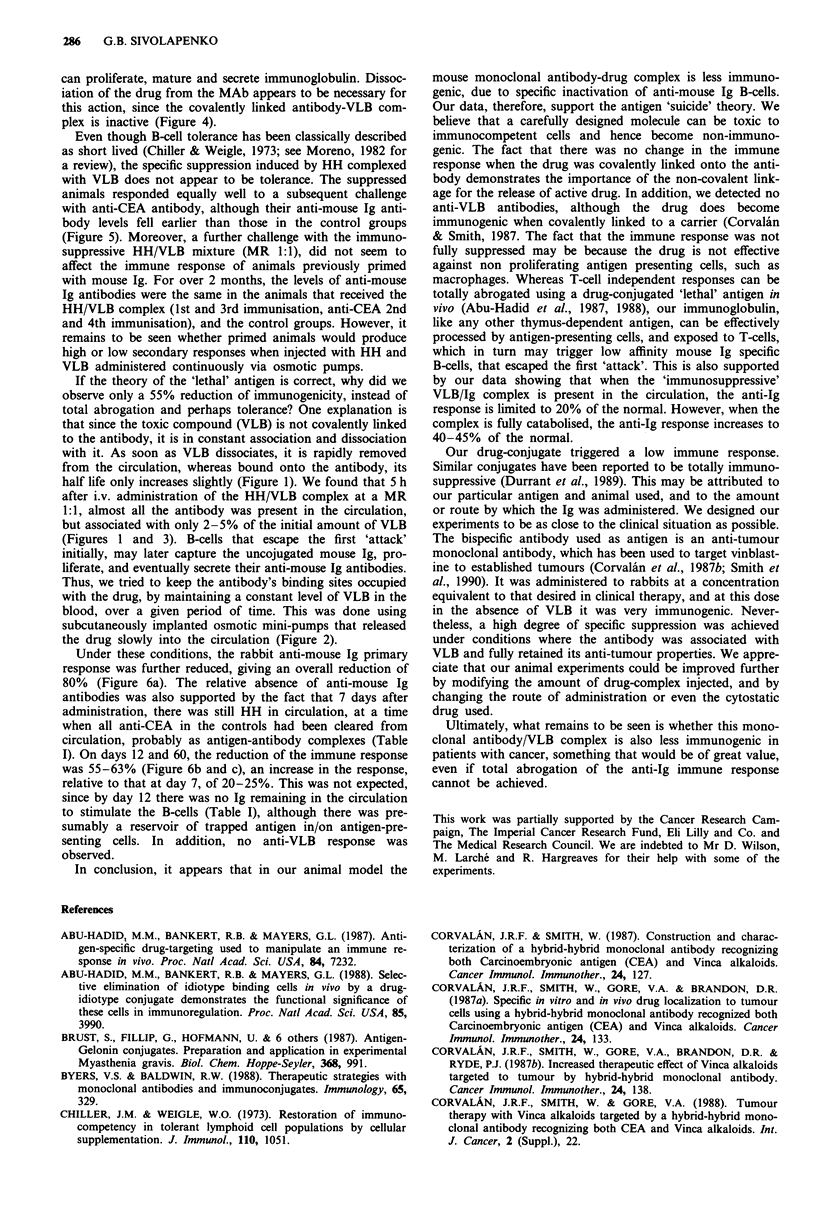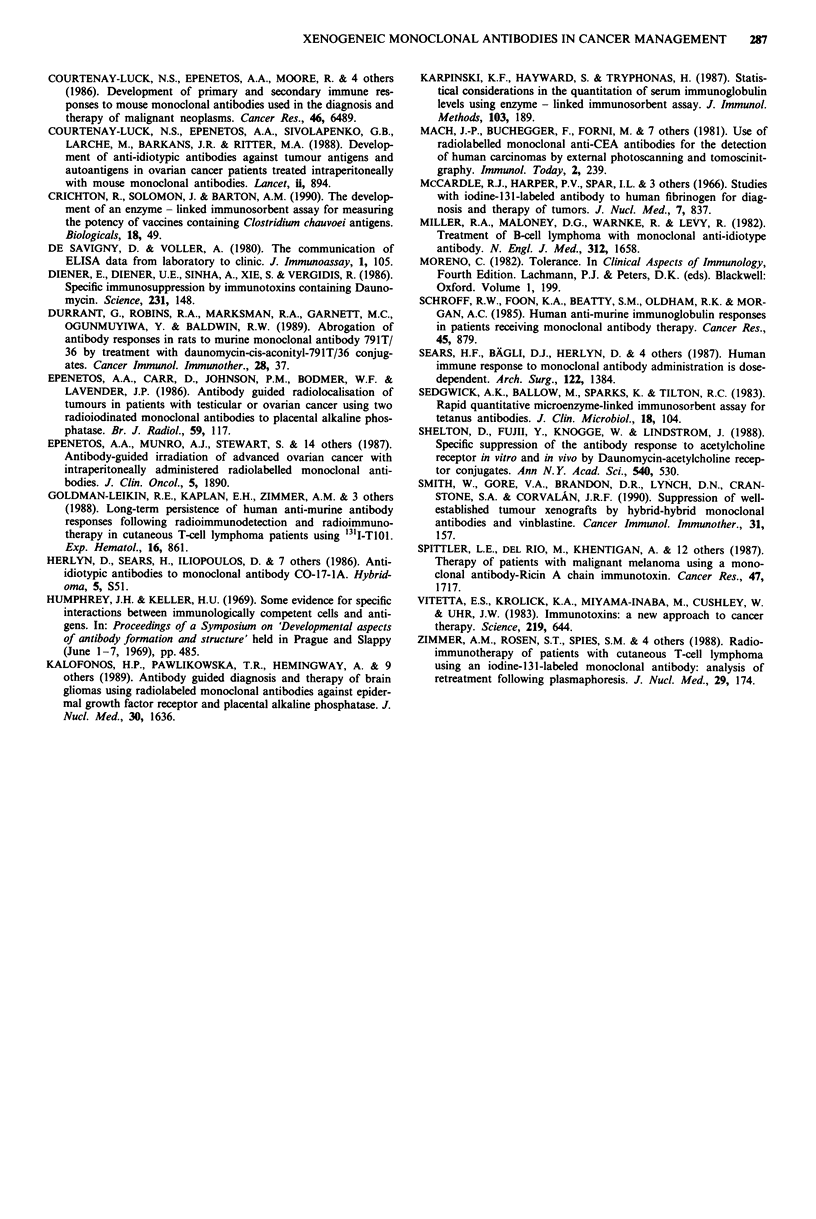# Xenogeneic monoclonal antibodies in the management of cancer: control of their in vivo immunogenicity and induction of specific unresponsiveness using an antibody-drug immunoconjugate.

**DOI:** 10.1038/bjc.1991.292

**Published:** 1991-08

**Authors:** G. B. Sivolapenko, C. Moreno, W. Smith, J. Corválan, M. A. Ritter, A. A. Epenetos

**Affiliations:** Department of Immunology, Royal Postgraduate Medical School, Hammersmith Hospital, London, UK.

## Abstract

A bispecific mouse monoclonal antibody (mAb) that recognises carcinoembryonic antigen (CEA) with one binding site and vinblastine (VLB) with the other was used, and its in vivo immunosuppressive effect specific for anti-mouse immunoglobulin (Ig) was studied. The antibody was incubated with VLB at a molar ratio (MR) of 1:1, and administered i.v. to rabbits. Control animals received either the MAb alone, or the MAb with VLB covalently linked (MR 1:1), or the parental anti-CEA with equimolar amount of VLB. Seven days later, the rabbit anti-mouse Ig primary response was measured, and found to be almost 55% reduced in the animals that received the VLB 'loaded' MAb. In vivo kinetics and stability experiments revealed that the T1/2 of the MAb was 68 +/- 5 h, whereas free VLB disappeared within minutes. It was concluded that as soon as the drug dissociates from the antibody's binding site, it is rapidly removed. This problem was overcome by subcutaneously implanting osmotic mini-pumps containing VLB. The pumps released the drug at a constant rate for a period greater than 1 week, saturating the antibody's binding site. Under these conditions rabbits developed 80% less anti-mouse Ig antibodies when the bispecific antibody was administered (compared with the parental anti-CEA). The immunosuppression observed was specific for the mouse Ig, under conditions compatible with the full clinical therapeutic potential of the MAb. In conclusion, these experiments show, that it is possible to develop hybrid antibodies that can act as a 'lethal bait' to any specific lymphocyte in vivo, thus preventing undesirable responses against the xenogeneic MAb.


Br. J. Cancer (1991), 64, 281-287 © Macmillan Press Ltd., 1991

### Xenogeneic monoclonal antibodies in the management of cancer: control of their *in vivo* immunogenicity and induction of specific unresponsiveness using an antibody-drug immunoconjugate

G.B. Sivolapenko[1,3], C. Moreno[2], W. Smith[4], J. Corválan[5], M.A. Ritter[1] & A.A. Epenetos[3]

[1]*Department of Immunology,* [2]*MRC Tuberculosis and Related Infections Unit, and* [3]*ICRF Oncology Group, Royal Postgraduate Medical School, Hammersmith Hospital, London, UK;* [4]*Lilly Research Centre Ltd., Eli Lilly and Co., Surrey, UK;* [5]*Hybritech Co., La Jolla, California, USA.*

**Summary** A bispecific mouse monoclonal antibody (mAb) that recognises carcinoembryonic antigen (CEA) with one binding site and vinblastine (VLB) with the other was used, and its *in vivo* immunosuppressive effect specific for anti-mouse immunoglobulin (Ig) was studied. The antibody was incubated with VLB at a molar ratio (MR) of 1:1, and administered i.v. to rabbits. Control animals received either the MAb alone, or the MAb with VLB covalently linked (MR 1:1), or the parental anti-CEA with equimolar amount of VLB. Seven days later, the rabbit anti-mouse Ig primary response was measured, and found to be almost 55% reduced in the animals that received the VLB 'loaded' MAb. *In vivo* kinetics and stability experiments revealed that the $T_{\frac{1}{2}}$ of the MAb was 68 ± 5 h, whereas free VLB disappeared within minutes. It was concluded that as soon as the drug dissociates from the antibody's binding site, it is rapidly removed. This problem was overcome by subcutaneously implanting osmotic mini-pumps containing VLB. The pumps released the drug at a constant rate for a period >1 week, saturating the antibody's binding site. Under these conditions rabbits developed 80% less anti-mouse Ig antibodies when the bispecific antibody was administered (compared with the parental anti-CEA). The immunosuppression observed was specific for the mouse Ig, under conditions compatible with the full clinical therapeutic potential of the MAb. In conclusion, these experiments show, that it is possible to develop hybrid antibodies that can act as a 'lethal bait' to any specific lymphocyte *in vivo*, thus preventing undesirable responses against the xenogeneic MAb.

Antibodies have been used in patients with cancer for *in vivo* tumour localisation and treatment, and encouraging results have been shown occasionally on early tumour detection (Mach *et al.*, 1981; Epenetos *et al.*, 1986; Kalofonos *et al.*, 1989) or effective cancer treatment (McCardle *et al.*, 1966; Miller *et al.*, 1982; Spittler *et al.*, 1987; Epenetos *et al.*, 1987; Byers & Baldwin, 1988). The introduction of the monoclonal antibody technique has played an important role in recent developments, since it enabled the production of a wide variety of monospecific antibodies in large quantities. Thus today, antibodies can be produced *in vitro*, and selected for properties that suit individual needs, such as affinity and isotype. In addition, progress in chemical modifications has enabled the production of novel immunoconjugates. Monoclonal antibodies (MAbs) can be conjugated to suitable radionuclide, enzymes, toxins (of plant or bacterial origin) and drugs, in order to increase their potency.

However, a major limitation to the successful *in vivo* clinical application of MAbs is that the majority of diagnostic and therapeutic antibodies are of rodent origin, and hence are recognised by the host as foreign. Patients can mount an immune response against the xenogeneic antibody (Schroff *et al.*, 1985; Goldman-Leikin *et al.*, 1988), which is dose dependent (Sears *et al.*, 1987), initially directed against the constant region (Courtenay-Luck *et al.*, 1986), but later extended against the whole molecule including the idiotope (Herlyn *et al.*, 1986; Courtenay-Luck *et al.*, 1988). Even if a successful treatment protocol is devised, the production of human anti-mouse immunoglobulin (Ig) antibodies can limit the efficiency of the administered therapeutic antibody by removing it from circulation (Zimmer *et al.*, 1988). As a consequence, the amount of the therapeutic antibody that could reach the tumour is very low, and continuously decreases upon further injections, as the anti-mouse Ig response rises. Furthermore, the *in vivo* antibody/antibody immune complexes may also lead to type III hypersensitivity (serum sickness), that can cause an inflammatory response with severe tissue damaging consequences.

The development of human anti-tumour antibodies, when readily available, may solve this problem, even though anti-idiotypic antibodies can be a source of difficulties. Thus, xenogeneic antibodies will continue to be in extensive use in a variety of clinical studies. It is therefore of paramount importance to reduce or abolish the patients' response against these rodent antibodies if we wish antibody-mediated manipulation of cancer to be successful.

The overall aim of this study was to identify ways by which the host's immune response to xenogeneic Ig, administered for therapeutic purposes, could be reduced or eliminated. We describe an approach towards the construction of Igs potentially 'lethal' to lymphocytes *in vivo*. Theoretically, if an antigen (the monoclonal antibody in this case) is conjugated to a toxic compound, it should kill rather than drive the patient's B-cells to differentiation and antibody secretion, in agreement with the concept of antigen 'suicide' first described by Humphrey & Keller, 1969. Thus, only the B-cell population that bears Ig receptors specific for the 'lethal' antigen will be deleted. Specific immunosuppression, and perhaps even tolerance, may be achieved under conditions where the rest of the immune system remains intact and fully potent to react against potential pathogens.

The bispecific antibody 28.19.8 recognises a tumour associated antigen (carcinoembrionic antigen, CEA) with one binding site and the Vinca alkaloids with the other (Corvalán & Smith, 1987). This antibody has been used successfully to target free, unmodified Vinca alkaloids to tumours expressing CEA, with significant suppression of tumour growth (Corvalán *et al.*, 1987*a*,*b*; Corvalán *et al.*, 1988; Smith *et al.*, 1990). In this study we have examined whether 28.19.8 (from here onwards called HH antibody) when 'loaded' with the cytostatic drug vinblastine (VLB) and administered to rabbits, could play the role of 'lethal' antigen, eliminate immunocompetent cells *in vivo*, and thus specifically suppress the anti-mouse Ig response. The results, if successful, could be of importance not only to immunotherapy, but also to other clinical situations such as autoimmunity, allergy or transplantation.

Correspondence: A.A. Epenetos, ICRF Oncology Group, Royal Postgraduate Medical School, Hammersmith Hospital, Ducane Road, London W12 0HS, UK.
Received 12 November 1990; and in revised form 7 February 1991.

### Materials and methods

### *Animals*

Male 1/2 'lop' rabbits (Froxfield Farm, UK), 3–4 kg, were used throughout this study.

### *Antigen*

The bispecific mouse monoclonal antibody 28.19.8. was used as immunogen for rabbits. This antibody was produced at the Lilly Research Centre, Eli Lilly and Co., Surrey, UK. It is a mouse IgG1-IgG2a hybrid, derived from the fusion of a hybridoma cell line producing anti-CEA monoclonal antibodies (designated as 11.285.14), and spleen cells from a mouse previously immunised with vindesine-bovine serum albumin (VDS-BSA). This hybrid-hybrid (HH) antibody designated as 28.19.8) has the capacity of binding CEA with one binding site and Vinca alkaloids with the other, the latter with an affinity constant of $\sim 5 \times 10^8 \ M^{-1}$ (unpublished data) as determined by equilibrium dialysis. It has been shown, that when 'loaded' with vindesine (Corvalán *et al.*, 1987*a*) or vinblastine (Corvalán *et al.*, 1987*b*), it is toxic to CEA expressing cells, not only *in vitro* but also *in vivo* against human tumour xenografts in nude mice (Corvalán *et al.*, 1988; Smith *et al.*, 1990).

Prior to each administration, the ability of the bispecific antibody to bind VLB was checked. This was done by either ELISA or gel filtration. For the ELISA, 96-well plates (Sterilin, UK) were coated with 200 ng well VLB-BSA; the test antibody was applied, followed by an anti-mouse Ig-horse-radish peroxidate (HRP, Amersham, UK) at 1:500 dilution. The substrate ABTS was applied, and the absorbance was measured at 405 nm (Titertek, Flow Laboratories, UK). For the gel filtration, the test antibody was incubated with $^3$H-labelled VLB ($^3$H-VLB, Amersham), and passed through Sephadex G-25 or G-50 columns (Pharmacia, Sweden). Fractions were collected, and the radioactivity was measured in a $\beta$-counter (LKB-Wallac, Sweden). Internal controls for quenching were introduced in each sample and the original counts corrected accordingly.

The immunoreactivity of the parental anti-CEA antibody was also tested by ELISA: Nunc (UK) 96-well plates were coated with 1 $\mu$g well purified CEA at room temperature (RT), overnight. The test antibody was added, followed by a rabbit anti-mouse Ig-alkaline phosphatase (prepared by Lilly Research Centre, Eli Lilly and Co., UK). The substrate was was p-nitro phenol phosphate (p-NPP, Sigma, UK), and the absorbance was measured at 410 nm, using a Titertek spectrophotometer.

### *Vinblastine-conjugates*

The hybrid-hybrid/VLB mixture was prepared by mixing the antibody with VLB sulphate (Eli Lilly, USA) for 15 min at RT. Prior to each administration all antibody and antibody-VLB mixtures were tested and found to be free of aggregates. This was done by size exclusion chromatography using a Superose 6 column connected to an FPLC system (Pharmacia).

Vinblastine was covalently linked to a bispecific antibody as follows: N-hydroxy succinimide ester of 4-succinoyl desacetyl vinblastine (Eli Lilly, USA) was allowed to bind to the bispecific antibody as antigen, whilst stirred for 1 min in phosphate buffered saline (PBS), pH 6.2, at 4°C. The starting MR was five vinblastine esters per antibody molecule. The antibody/vinblastine ester complex was immediately separated from free ester by size exclusion chromatography (P-6 column, Biorad, UK) in borate buffer, pH 8.6, at 4°C. The complex was warmed to RT, and stirred for 1 h. After overnight dialysis in PBS, pH 7.4, the conjugation ratio was estimated by dual wavelength spectroscopy at 270 and 280 nm, and found to be 1.03 VLB/antibody. As tested by ELISA, the immunoreactivity of the conjugate for CEA remained intact, whereas it was reduced to 2.7% for VLB, when compared with the unconjugated antibody.

Similarly, VLB was conjugated to BSA using the same succinamide ester as above. The mixture was incubated in borate buffer, pH 8.6, for 4 h at RT, and BSA-VLB was purified from unbound VLB ester by gel filtration (P-6 column, Biorad) in PBS, pH 7.4. The MR was found to be 3.75:1 (starting MR 15:1).

### *Kinetics*

The presence of vinblastine was measured in rabbits when administered i.v., when constantly infused using a mini osmotic pump designed to deliver 10 $\mu l \ h^{-1}$ for a week (Alzet Co., Palo Alto, USA) implanted subcutaneously, and when co-injected i.v. with the bispecific antibody. Prior to administration the drug was mixed with a trace amount of $^3$H-VLB (Amersham), blood samples were taken at regular time points, and the radioactivity in the serum was measured using a $\beta$-counter.

The kinetics of the bispecific monoclonal antibody, when administered i.v. to rabbits, were measured as follows: the antibody was injected, and blood samples were taken at various intervals for 5 days. The blood was left to clot, the serum was then removed and incubated in 96-well plates (Sterilin) coated with 200 ng well VLB-BSA. After 2 h the plate was washed with PBS/0.05% tween 20, pH 7.4, and incubated with an anti-mouse Ig-HRP (Amersham) at 1:500 dilution, for 1 h. The plate was washed, and the absorbance was measured at 405 nm using azino-di-[3-ethylbenzothiazo-linesulphonate] (ABTS) substrate. The results were given as areas under the sigmoidal curve, obtained from the serial dilutions (1:10) of the test sera. The value obtained from the serum sample taken immediately after administration was regarded as the injected dose (100%).

### *Immunisation protocols*

The immunisation schedule of the first experiment was as follows: four groups of three rabbits each were injected i.v. (ear vein) with 1 mg of the following antigens: (a) the bispecific, hybrid-hybrid, mouse monoclonal antibody 28.19.8 mixed with VLB (as VLB sulphate, Eli Lilly, USA) (HH/VLB) at a molar ratio (MR) of 1:1 (equivalent to 5.45 $\mu$g VLB/rabbit), (b) the parental anti-CEA antibody 11.285.14 mixed with 5.45 $\mu$g VLB (anti-CEA/VLB), (c) the bispecific monoclonal antibody 28.19.8 alone (HH) and lastly (d) the bispecific monoclonal antibody 28.19.8 covalently linked to VLB (HH-c-VLB), on the Vinca binding site, at MR of 1:1. All rabbits were bled on day 6, and boosted i.p. with 1 mg of the parental anti-CEA antibody on day 7. They were bled again on days 10, 14, 21 and 28. On day 29 the four groups received i.v. 1 mg of the initial immunogen as on day 0. A blood sample was taken 7 days later (day 35), and on day 36, all rabbits were boosted again i.p. with 1 mg of the anti-CEA (as on day 7). They were bled on days 39, 43 and from then on, once weekly for a period of 3 weeks (up to day 64). In addition, 4 mg of ovalbumin (OVA) (Sigma, UK) was simultaneously administered with each injection of the antigen.

The second experiment consisted of two groups of six rabbits each. In all animals, an osmotic mini-pump of the same characteristics as described above and filled with 1 mg of VLB in distilled water was implanted subcutaneously in the dorsal region between the scapulae. Five days after implantation, the two groups received i.v. 1 mg of either the bispecific antibody (HH) or the parental anti-CEA. The animals were bled, and the primary anti-mouse Ig response assessed on days 7, 12 and 60 after administration of the immunogen.

### *Measurement of circulating immunoglobin and vinblastine*

The concentration of the mouse antibodies (used as immunogens i.e. HH or anti-CEA) in the circulation was measured by ELISA. Ninety-six well microtitre plates (Nunc) were coated with 1 $\mu$g well purified CEA. Test serum was added, and the mouse antibodies were detected by a rabbit antimouse Ig-alkaline phosphatase conjugate and p-NPP substrate. The absorbance was measured at 410 nm, by a multiscan spectrophotometer, a standard curve plotted, using known concentrations of the corresponding antibody (mixed with rabbit serum), and the circulating immunogen quantitated.

Similarly the concentration of circulating VLB was estimated as previously described (Corvalán & Smith, 1987): ELISA plates were coated with CEA (as above), and a saturating amount of the bispecific antibody was incubated for 1 h at RT. The plates were washed with PBS/0.05% Tween 20, pH 7.4, and the test serum was added. Circulating VLB was detected, by direct competition with VLB-alkaline phosphatase. The substrate used was p-NPP, and the absorbance was measured at 410 nm as above. This was performed after it was established that there was no HH antibody in circulation to interfer with our assay. The assay is sensitive to less than 1 ng VLB $ml^{-1}$.

### *Measurement of the anti-immunoglobulin response*

The rabbits' responses against the mouse monoclonal antibodies administered were measured by ELISA. Ninety-six well plates were coated with the antibody used as antigen, and the corresponding test sera were added at 10-fold dilutions and tested in duplicates. Rabbit anti-mouse Ig antibodies were detected by a species-specific anti-mouse Ig-HRP second layer and ABTS substrate. After plotting the optical densities *vs* the serum dilutions, the results were analysed by a weighted non linear least squares of 4 parameters logit (De Savigny & Voller, 1980; Karpinski *et al.*, 1987). The results were expressed as areas under the serum titration sigmoidal curve as this method has been duly validated before (Sedgwick *et al.*, 1983; Crichton *et al.*, 1990). Before any experiment, it was ensured that none of the rabbits used had any pre-existing anti-mouse Ig antibodies. Statistical analysis of the data was performed using the Student's *t*-test on the logs of the areas under the titration curves.

### *Measurement of the anti-ovalbumin and anti-vinblastine response*

The rabbits' response to the administered VLB or OVA was measured with an ELISA similar to the above. The only difference was that the plates were coated with either 200 ng well VLB-BSA or 500 ng well OVA. In the case of anti-VLB response, all positive sera were also tested against BSA alone, in order to prove the presence of true anti-VLB antibodies. Before each experiment, rabbits were chosen that did not have any pre-existing anti-VLB or anti-OVA antibodies. Statistical analysis of the data was also performed using the Student's *t*-test on the logs of the area.

### Results

### *Kinetics of vinblastine*

The kinetics of free VLB in rabbits were measured after i.v. administration. Serum samples were taken at various time points up to 15 min. The drug was rapidly removed from the circulation; 10 min after administration approximately 1% of the injected dose remained in the blood (Figure 1). The clearance of VLB when simultaneously administered with the hybrid-hybrid antibody was also measured over a period of 5 h. When VLB was injected i.v. into a rabbit in the presence of HH at a Mr of 1:1, the drug was rapidly removed from the circulation, though not as rapidly as free VLB. As shown in Figure 1, at 10 min after administration approximately 15% of the injected amount of VLB was in circulation associated with the HH antibody.

In addition, the kinetics of free VLB were studied after infusion from a subcutaneously implanted osmotic mini-pump. The pump was filled with VLB and the animal was bled daily for up to 20 days. It was found that the drug was released slowly into the circulation, and as shown in Figure 2, its concentration increased with time. The amount of VLB reached a peak 7–9 days after implantation, after which it slowly dropped back to zero (Figure 2). It was estimated that from day 5 to day 12 there was a 'window', where the total amount of VLB in the blood was over 5 μg, i.e. a MR of approximately 1:1 when 1 mg of HH is injected; amount sufficient to saturate the MAb.

### *Kinetics of the monoclonal antibody*

The hybrid-hybrid antibody was injected i.v. into a rabbit, and serum samples were collected at various time points for 5 days. The amount of antibody present in circulation at each time point was determined by its ability to bind VLB immunobilised onto an ELISA plate. The amount of HH present in the blood – and also capable of binding to its antigen – remained unchanged (100% of the injected dose) for up to 6 h after administration. From that point on, it diminished with a $T_{\frac{1}{2}}$ of 68 ± 5 h (Figure 3).

**Figure 1** Clearance of vinblastine in rabbits, after i.v. administration. □, free vinblastine; ■, vinblastine in the presence of the hybrid-hybrid antibody.

**Figure 2** Total concentration of vinblastine in the serum of rabbits when the drug is constantly infused by a subcutaneously implanted osmotic mini-pump.

**Figure 3** Clearance of the bispecific antibody in rabbits after i.v. administration.

*Anti-mouse immunoglobulin response*

In the first experiment, three rabbits/group were primed i.v. with the HH, HH covalently conjugated with VLB, HH mixed with VLB and anti-CEA mixed with VLB (the last three preparations were used at a MR of 1:1). When the primary anti-mouse Ig response was measured 7 days later, we found that rabbits receiving the HH/VLB mixture responded significantly less to the mouse Ig. As shown in Figure 4, rabbits primed with the VLB- 'loaded' HH, developed 53.4% less anti-mouse Ig antibodies, than those that received HH alone ($Mean_{HH/VLB} = 1.368 \pm 0.370$, $Mean_{HH} = 2.932 \pm 0.304$, $P = 0.004$), 52.4% less than those receiving the HH antibody covalently linked to VLB (Mean = HH-c-VLB = $2.875 \pm 0.407$, $P = 0.006$) and 59.8% less than those receiving the parental anti-CEA antibody mixed with VLB ($Mean_{a\text{-}CEA/VLB} = 3.401 \pm 0.018$). Only two animals were available for comparison in this last control group. The titres of these two animals were very similar to those in the other control groups injected with HH alone and HH covalently coupled to VLB, respectively.

On day 7, all groups were boosted i.p. with the anti-CEA antibody. As shown in Figure 5, the anti-mouse Ig secondary response was measured over a period of 3 weeks. We found that initially there was no difference in the response among the four groups. Nevertheless, the anti-mouse Ig titres started to drop more rapidly (on day 21) in the group of rabbits that were primed with the HH/VLB mixture. By day 28, the anti-mouse Ig response for this group (mean $2.010 \pm 0.114$) was 32% less ($P = 0.021$) than those receiving the HH antibody alone (mean $2.950 \pm 0.180$), 23% less ($P = 0.035$) than those receiving HH antibody covalently linked to VLB (mean $2.609 \pm 0.088$) and 28% less ($P = 0.004$) than those receiving the parental anti-CEA antibody mixed with VLB (mean $2.780 \pm 0.056$). The complete results are presented in Figure 5.

On day 29, the four groups received an i.v. injection of their corresponding antigens (Igs) (as on day 0), and 7 days later they were boosted with an i.p. injection of the anti-CEA antibody alone (as on day 7). The humoral response was followed up to day 64; we observed no difference in the rabbits' anti-mouse Ig responses among all groups, irrespective of the preparations with which they had been primed and boosted.

In the second experiment, 12 rabbits were implanted with osmotic mini-pumps, releasing VLB into the circulation at a constant flow rate. Five days after implantation, six rabbits received the HH antibody and the other six the parental anti-CEA. The rabbits' primary reponse was measured on days 7, 12 and 60 post antibody administration. As shown in Figure 6a, the response of rabbits injected with the HH had a 78% lower response than in the group receiving the control antibody ($Mean_{HH} = 0.480 \pm 0.237$, $Mean_{a\text{-}CEA} = 2.177 \pm 0.802$, $P < 0.001$). On days 12 and 60, the reduction in the anti-mouse Ig response was found to be 55% ($Mean_{HH} = 1.799 \pm 0.614$, $Mean_{a\text{-}CEA} = 3.587 \pm 0.463$, $P = 0.001$) and 63% ($Mean_{HH} = 1.128 \pm 0.345$, $Mean_{a\text{-}CEA} = 3.007 \pm 0.941$, $P < 0.001$) respectively (Figure 6b and c). The overall anti-mouse Ig response, over the period of 60 days, is shown in Figure 7. The mean reduction in the anti-mouse Ig response for the rabbits that received the bispecific antibody, was 60%, as calculated by the area under the curve.

For this experiment, where the drug was delivered by an osmotic mini-pump, we measured the levels of HH and anti-CEA still in circulation, on day 7 and 12 after administration. As shown in Table I, five our of the six rabbits that received the HH antibody (and responded significantly less to it), still had detectable levels of the MAb in circulation. The 6th rabbit was the one that developed the highest anti-mouse Ig response of this group (Figure 6a). In addition, the five out of six rabbits that were injected with the anti-CEA antibody (and responded very well to it), we did not detect any anti-CEA in the circulation. In only one rabbit (number 7) could we detect circulating mouse Ig, and that was the one that developed the lowest anti-mouse Ig response of this group (Figure 6a). By day 12 there was no mouse immunoglobutin in circulation. The VLB levels in the blood were also measured on day 12. As shown in Table I, there was still VLB in circulation. There appeared to be no relationship between the kind of immunogen administered and the amount of circulating VLB.

**Figure 4** Rabbit anti-mouse Ig primary response, when the following immunogens were administered i.v. in groups of three rabbits: the hybrid-hybrid antibody (HH), the hybrid-hybrid antibody covalently linked to vinblastine (HH-c-VLB), the anti-CEA antibody mixed with vinblastine (a-CEA/VLB) and the hybrid-hybrid antibody mixed with vinblastine (HH/VLB). The a-CEA/VLB group consisted of only two rabbits.

**Figure 5** Rabbit anti-mouse Ig secondary response, measured over a period of 4 weeks, when four groups of rabbits were primed i.v. (day 0) with: □, the hybrid-hybrid antibody (HH), ■, the hybrid-hybrid antibody covalently linked to vinblastine (HH-c-VLB), ◆, the anti-CEA antibody mixed with vinblastine (a-CEA/ VLB) and ◇, the hybrid-hybrid antibody mixed with vinblastine, and subsequently boosted i.p. (day 7) with the a-CEA antibody.

*Anti-ovalbumin and anti-vinblastine response*

Rabbits were also immunised with OVA as an irrelevant antibody. The anti-OVA response was followed throughout the experiments indicating that, despite variability, there was no significant difference in the animals' ability to make antibodies against OVA, irrespective of the immunoglobulin preparation that they had simultaneously received (Table II). All animals were also tested for an anti-VLB response. No anti-VLB antibodies were observed under the immunisation conditions used.

*Toxicity*

All animals remained alive and well throughout and long after the experiments. No side effects were observed at the doses of vinblastine and mouse antibodies that were administered, and no adverse reactions were seen due to the implantation of the mini-pump, apart from a transient and self-limiting erythema.

**Figure 6** Primary anti-mouse Ig response, when the hybrid-hybrid and the a-CEA antibodies were administered iv. to two groups of six rabbits, under constant infusion of vinblastine. The response was measured on days 7 **a**, 12 **b** and 60 **c** post administration of the antibodies.

**Figure 7** Mean overall primary anti-mouse Ig response, when the hybrid-hybrid (HH) and the a-CEA antibodies were administered i.v. to two groups of six rabbits, under constant infusion of vinblastine.

**Table I** Concentration of the hybrid-hybrid (HH) and the anti-CEA (a-CEA) antibodies in rabbits' sera, 7 and 12 days after i.v. administration

| | | *Antibody* | | *Vinblastine* | |
|---|---|---|---|---|---|
| *Rabbit* | *Immunogen* | *Day 7 (ng ml$^{-1}$)* | *Day 12 (ng ml$^{-1}$)* | *Day 7 (ng ml$^{-1}$)* | *Day 12 (ng ml$^{-1}$)* |
| 1 | HH | 108 | – | N.T. | 4.5 |
| 2 | | 237 | – | N.T. | <0.4 |
| 3 | | 83 | – | N.T. | 4.3 |
| 4 | | 26 | – | N.T. | N.T. |
| 5 | | 77 | – | N.T. | <0.4 |
| 6 | | – | – | N.T. | 7.8 |
| 7 | a-CEA | 124 | – | N.T. | <0.2 |
| 8 | | – | – | N.T. | N.T. |
| 9 | | – | – | N.T. | 1.3 |
| 10 | | – | – | N.T. | <0.13 |
| 11 | | – | – | N.T. | 27 |
| 12 | | N.T. | N.T. | N.T. | 1.3 |

Concentration of vinblastine in rabbit's sera at the same time as above. The drug was constantly infused into the rabbits by an osmotic mini-pump, subcutaneously implanted 5 days before administration of the antibodies.

**Table II** Anti-ovalbumin (OVA) response for four groups of rabbits primed with OVA on day 0 in combination with either HH antibody, HH-c-VLB, a-CEA/VLB, or HH/VLB, respectively

| *Day* | *HH* | *HH-c-VLB* | *a-CEA/VLB* | *HH/VLB* |
|---|---|---|---|---|
| 6 | 0.265±0.063 | 0.149±0.016 | 0.183 | 0.265±0.060 |
| 10 | 1.169±0.494 | 0.555±0.331 | 0.640 | 1.195±0.401 |
| 14 | 1.992±0.742 | 1.672±0.349 | 1.453 | 1.590±0.421 |
| 21 | 1.602±0.531 | 1.395±0.392 | 1.285 | 1.412±0.421 |
| 28 | 1.313±0.339 | 1.220±0.350 | 1.127 | 1.051±0.292 |

All rabbits were rechallenged with the same immunogens after 7 days. *Only two animals in this group. *P* values were $>0.1$ for days 6 and 10 and $>0.5$ for all the rest.

### Discussion

It has previously been shown by many investigators that when an antigen is covalently linked to plant toxins or drugs, it can specifically inactivate lymphocytes that bind to it *in vitro* (Vitetta *et al.*, 1983; Shelton *et al.*, 1988). Moreover, in certain cases antigen-toxin/drug conjugates were found to abrogate specifically the response of animals against the administered antigen (Shelton *et al.*, 1988; Brust *et al.*, 1987; Diener *et al.*, 1986; Durrant *et al.*, 1989). We have used a novel drug-conjugate consisting of a bispecific mouse monoclonal antibody, that recognises Vinca alkaloids with one binding site and CEA with the other. This hybrid-hybrid antibody when mixed with vinblastine, forms a complex that is stable *in vitro*, as it was estimated by gel filtration (see Methods), even in the presence of serum. We investigated the possible *in vivo* immunosuppressive effects of this monoclonal antibody/VLB complex, when it was administered 'loaded' with vinblastine to rabbits.

We found that when this bispecific monoclonal antibody was administered i.v. to rabbits simultaneously with VLB, at a MR of 1:1, the primary anti-mouse Ig response was reduced by approximately 55% (Figure 4). The immunosuppression observed was specific for the mouse Ig, since the response against an irrelevant antigen (OVA) was normal (Table II). Further proof that the amount of VLB injected was not responsible for a general, non specific immunosuppression, was that when the drug was co-injected with the control anti-CEA antibody, the anti-mouse Ig response was not reduced (Figure 4). Interestingly, when the hybrid-hybrid antibody was covalently linked to VLB, it did not show any immunosuppressive effect (Figure 4). It would therefore appear, that the hybrid-hybrid/VLB complex is captured by B-cells that have receptors specific for the mouse Ig administered. The VLB subsequently dissociates and inactivates the mouse Ig-specific B-cells, which eventually die before they can proliferate, mature and secrete immunoglobulin. Dissociation of the drug from the MAb appears to be necessary for this action, since the covalently linked antibody-VLB complex is inactive (Figure 4).

Even though B-cell tolerance has been classically described as short lived (Chiller & Weigle, 1973; see Moreno, 1982 for a review), the specific suppression induced by HH complexed with VLB does not appear to be tolerance. The suppressed animals responded equally well to a subsequent challenge with anti-CEA antibody, although their anti-mouse Ig antibody levels fell earlier than those in the control groups (Figure 5). Moreover, a further challenge with the immunosuppressive HH/VLB mixture (MR 1:1), did not seem to affect the immune response of animals previously primed with mouse Ig. For over 2 months, the levels of anti-mouse Ig antibodies were the same in the animals that received the HH/VLB complex (1st and 3rd immunisation, anti-CEA 2nd and 4th immunisation), and the control groups. However, it remains to be seen whether primed animals would produce high or low secondary responses when injected with HH and VLB administered continuously via osmotic pumps.

If the theory of the 'lethal' antigen is correct, why did we observe only a 55% reduction of immunogenicity, instead of total abrogation and perhaps tolerance? One explanation is that since the toxic compound (VLB) is not covalently linked to the antibody, it is in constant association and dissociation with it. As soon as VLB dissociates, it is rapidly removed from the circulation, whereas bound onto the antibody, its half life only increases slightly (Figure 1). We found that 5 h after i.v. administration of the HH/VLB complex at a MR 1:1, almost all the antibody was present in the circulation, but associated with only 2–5% of the initial amount of VLB (Figures 1 and 3). B-cells that escape the first 'attack' initially, may later capture the uncojugated mouse Ig, proliferate, and eventually secrete their anti-mouse Ig antibodies. Thus, we tried to keep the antibody's binding sites occupied with the drug, by maintaining a constant level of VLB in the blood, over a given period of time. This was done using subcutaneously implanted osmotic mini-pumps that released the drug slowly into the circulation (Figure 2).

Under these conditions, the rabbit anti-mouse Ig primary response was further reduced, giving an overall reduction of 80% (Figure 6a). The relative absence of anti-mouse Ig antibodies was also supported by the fact that 7 days after administration, there was still HH in circulation, at a time when all anti-CEA in the controls had been cleared from circulation, probably as antigen-antibody complexes (Table I). On days 12 and 60, the reduction of the immune response was 55–63% (Figure 6b and c), an increase in the response, relative to that at day 7, of 20–25%. This was not expected, since by day 12 there was no Ig remaining in the circulation to stimulate the B-cells (Table I), although there was presumably a reservoir of trapped antigen in/on antigen-presenting cells. In addition, no anti-VLB response was observed.

In conclusion, it appears that in our animal model the mouse monoclonal antibody-drug complex is less immunogenic, due to specific inactivation of anti-mouse Ig B-cells. Our data, therefore, support the antigen 'suicide' theory. We believe that a carefully designed molecule can be toxic to immunocompetent cells and hence become non-immunogenic. The fact that there was no change in the immune response when the drug was covalently linked onto the antibody demonstrates the importance of the non-covalent linkage for the release of active drug. In addition, we detected no anti-VLB antibodies, although the drug does become immunogenic when covalently linked to a carrier (Corvalán & Smith, 1987. The fact that the immune response was not fully suppressed may be because the drug is not effective against non proliferating antigen presenting cells, such as macrophages. Whereas T-cell independent responses can be totally abrogated using a drug-conjugated 'lethal' antigen *in vivo* (Abu-Hadid *et al.*, 1987, 1988), our immunoglobulin, like any other thymus-dependent antigen, can be effectively processed by antigen-presenting cells, and exposed to T-cells, which in turn may trigger low affinity mouse Ig specific B-cells, that escaped the first 'attack'. This is also supported by our data showing that when the 'immunosuppressive' VLB/Ig complex is present in the circulation, the anti-Ig response is limited to 20% of the normal. However, when the complex is fully catabolised, the anti-Ig response increases to 40–45% of the normal.

Our drug-conjugate triggered a low immune response. Similar conjugates have been reported to be totally immunosuppressive (Durrant *et al.*, 1989). This may be attributed to our particular antigen and animal used, and to the amount or route by which the Ig was administered. We designed our experiments to be as close to the clinical situation as possible. The bispecific antibody used as antigen is an anti-tumour monoclonal antibody, which has been used to target vinblastine to established tumours (Corvalán *et al.*, 1987*b*; Smith *et al.*, 1990). It was administered to rabbits at a concentration equivalent to that desired in clinical therapy, and at this dose in the absence of VLB it was very immunogenic. Nevertheless, a high degree of specific suppression was achieved under conditions where the antibody was associated with VLB and fully retained its anti-tumour properties. We appreciate that our animal experiments could be improved further by modifying the amount of drug-complex injected, and by changing the route of administration or even the cytostatic drug used.

Ultimately, what remains to be seen is whether this monoclonal antibody/VLB complex is also less immunogenic in patients with cancer, something that would be of great value, even if total abrogation of the anti-Ig immune response cannot be achieved.

This work was partially supported by the Cancer Research Campaign, The Imperial Cancer Research Fund, Eli Lilly and Co. and The Medical Research Council. We are indebted to Mr D. Wilson, M. Larché and R. Hargreaves for their help with some of the experiments.

### References

ABU-HADID, M.M., BANKERT, R.B. & MAYERS, G.L. (1987). Antigen-specific drug-targeting used to manipulate an immune response *in vivo*. *Proc. Natl Acad. Sci. USA*, **84**, 7232.

ABU-HADID, M.M., BANKERT, R.B. & MAYERS, G.L. (1988). Selective elimination of idiotype binding cells *in vivo* by a drug-idiotype conjugate demonstrates the functional significance of these cells in immunoregulation. *Proc. Natl Acad. Sci. USA*, **85**, 3990.

BRUST, S., FILLIP, G., HOFMANN, U. & 6 others (1987). Antigen-Gelonin conjugates. Preparation and application in experimental Myasthenia gravis. *Biol. Chem. Hoppe-Seyler*, **368**, 991.

BYERS, V.S. & BALDWIN, R.W. (1988). Therapeutic strategies with monoclonal antibodies and immunoconjugates. *Immunology*, **65**, 329.

CHILLER, J.M. & WEIGLE, W.O. (1973). Restoration of immunocompetency in tolerant lymphoid cell populations by cellular supplementation. *J. Immunol.*, **110**, 1051.

CORVALÁN, J.R.F. & SMITH, W. (1987). Construction and characterization of a hybrid-hybrid monoclonal antibody recognizing both Carcinoembryonic antigen (CEA) and Vinca alkaloids. *Cancer Immunol. Immunother.*, **24**, 127.

CORVALÁN, J.R.F., SMITH, W., GORE, V.A. & BRANDON, D.R. (1987*a*). Specific *in vitro* and *in vivo* drug localization to tumour cells using a hybrid-hybrid monoclonal antibody recognized both Carcinoembryonic antigen (CEA) and Vinca alkaloids. *Cancer Immunol. Immunother.*, **24**, 133.

CORVALÁN, J.R.F., SMITH, W., GORE, V.A., BRANDON, D.R. & RYDE, P.J. (1987*b*). Increased therapeutic effect of Vinca alkaloids targeted to tumour by hybrid-hybrid monoclonal antibody. *Cancer Immunol. Immunother.*, **24**, 138.

CORVALÁN, J.R.F., SMITH, W. & GORE, V.A. (1988). Tumour therapy with Vinca alkaloids targeted by a hybrid-hybrid monoclonal antibody recognizing both CEA and Vinca alkaloids. *Int. J. Cancer*, **2** (Suppl.), 22.

COURTENAY-LUCK, N.S., EPENETOS, A.A., MOORE, R. & 4 others (1986). Development of primary and secondary immune responses to mouse monoclonal antibodies used in the diagnosis and therapy of malignant neoplasms. *Cancer Res.*, **46**, 6489.

COURTENAY-LUCK, N.S., EPENETOS, A.A., SIVOLAPENKO, G.B., LARCHE, M., BARKANS, J.R. & RITTER, M.A. (1988). Development of anti-idiotypic antibodies against tumour antigens and autoantigens in ovarian cancer patients treated intraperitoneally with mouse monoclonal antibodies. *Lancet*, **ii**, 894.

CRICHTON, R., SOLOMON, J. & BARTON, A.M. (1990). The development of an enzyme – linked immunosorbent assay for measuring the potency of vaccines containing *Clostridium chauvoei* antigens. *Biologicals*, **18**, 49.

DE SAVIGNY, D. & VOLLER, A. (1980). The communication of ELISA data from laboratory to clinic. *J. Immunoassay*, **1**, 105.

DIENER, E., DIENER, U.E., SINHA, A., XIE, S. & VERGIDIS, R. (1986). Specific immunosuppression by immunotoxins containing Daunomycin. *Science*, **231**, 148.

DURRANT, G., ROBINS, R.A., MARKSMAN, R.A., GARNETT, M.C., OGUNMUYIWA, Y. & BALDWIN, R.W. (1989). Abrogation of antibody responses in rats to murine monoclonal antibody 791T/36 by treatment with daunomycin-cis-aconityl-791T/36 conjugates. *Cancer Immunol. Immunother.*, **28**, 37.

EPENETOS, A.A., CARR, D., JOHNSON, P.M., BODMER, W.F. & LAVENDER, J.P. (1986). Antibody guided radiolocalisation of tumours in patients with testicular or ovarian cancer using two radioiodinated monoclonal antibodies to placental alkaline phosphatase. *Br. J. Radiol.*, **59**, 117.

EPENETOS, A.A., MUNRO, A.J., STEWART, S. & 14 others (1987). Antibody-guided irradiation of advanced ovarian cancer with intraperitoneally administered radiolabelled monoclonal antibodies. *J. Clin. Oncol.*, **5**, 1890.

GOLDMAN-LEIKIN, R.E., KAPLAN, E.H., ZIMMER, A.M. & 3 others (1988). Long-term persistence of human anti-murine antibody responses following radioimmunodetection and radioimmunotherapy in cutaneous T-cell lymphoma patients using $^{131}$I-T101. *Exp. Hematol.*, **16**, 861.

HERLYN, D., SEARS, H., ILIOPOULOS, D. & 7 others (1986). Anti-idiotypic antibodies to monoclonal antibody CO-17-1A. *Hybridoma*, **5**, S51.

HUMPHREY, J.H. & KELLER, H.U. (1969). Some evidence for specific interactions between immunologically competent cells and antigens. In: *Proceedings of a Symposium on 'Developmental aspects of antibody formation and structure'* held in Prague and Slappy (June 1–7, 1969), pp. 485.

KALOFONOS, H.P., PAWLIKOWSKA, T.R., HEMINGWAY, A. & 9 others (1989). Antibody guided diagnosis and therapy of brain gliomas using radiolabeled monoclonal antibodies against epidermal growth factor receptor and placental alkaline phosphatase. *J. Nucl. Med.*, **30**, 1636.

KARPINSKI, K.F., HAYWARD, S. & TRYPHONAS, H. (1987). Statistical considerations in the quantitation of serum immunoglobulin levels using enzyme – linked immunosorbent assay. *J. Immunol. Methods*, **103**, 189.

MACH, J.-P., BUCHEGGER, F., FORNI, M. & 7 others (1981). Use of radiolabelled monoclonal anti-CEA antibodies for the detection of human carcinomas by external photoscanning and tomoscintigraphy. *Immunol. Today*, **2**, 239.

McCARDLE, R.J., HARPER, P.V., SPAR, I.L. & 3 others (1966). Studies with iodine-131-labeled antibody to human fibrinogen for diagnosis and therapy of tumors. *J. Nucl. Med.*, **7**, 837.

MILLER, R.A., MALONEY, D.G., WARNKE, R. & LEVY, R. (1982). Treatment of B-cell lymphoma with monoclonal anti-idiotype antibody. *N. Engl. J. Med.*, **312**, 1658.

MORENO, C. (1982). Tolerance. In *Clinical Aspects of Immunology*, Fourth Edition. Lachmann, P.J. & Peters, D.K. (eds). Blackwell: Oxford. Volume 1, 199.

SCHROFF, R.W., FOON, K.A., BEATTY, S.M., OLDHAM, R.K. & MORGAN, A.C. (1985). Human anti-murine immunoglobulin responses in patients receiving monoclonal antibody therapy. *Cancer Res.*, **45**, 879.

SEARS, H.F., BÄGLI, D.J., HERLYN, D. & 4 others (1987). Human immune response to monoclonal antibody administration is dose-dependent. *Arch. Surg.*, **122**, 1384.

SEDGWICK, A.K., BALLOW, M., SPARKS, K. & TILTON, R.C. (1983). Rapid quantitative microenzyme-linked immunosorbent assay for tetanus antibodies. *J. Clin. Microbiol.*, **18**, 104.

SHELTON, D., FUJII, Y., KNOGGE, W. & LINDSTROM, J. (1988). Specific suppression of the antibody response to acetylcholine receptor *in vitro* and *in vivo* by Daunomycin-acetylcholine receptor conjugates. *Ann N.Y. Acad. Sci.*, **540**, 530.

SMITH, W., GORE, V.A., BRANDON, D.R., LYNCH, D.N., CRANSTONE, S.A. & CORVALÁN, J.R.F. (1990). Suppression of well-established tumour xenografts by hybrid-hybrid monoclonal antibodies and vinblastine. *Cancer Immunol. Immunother.*, **31**, 157.

SPITTLER, L.E., DEL RIO, M., KHENTIGAN, A. & 12 others (1987). Therapy of patients with malignant melanoma using a monoclonal antibody-Ricin A chain immunotoxin. *Cancer Res.*, **47**, 1717.

VITETTA, E.S., KROLICK, K.A., MIYAMA-INABA, M., CUSHLEY, W. & UHR, J.W. (1983). Immunotoxins: a new approach to cancer therapy. *Science*, **219**, 644.

ZIMMER, A.M., ROSEN, S.T., SPIES, S.M. & 4 others (1988). Radioimmunotherapy of patients with cutaneous T-cell lymphoma using an iodine-131-labeled monoclonal antibody: analysis of retreatment following plasmaphoresis. *J. Nucl. Med.*, **29**, 174.